# Primary Conjunctival Basal Cell Carcinoma Mimicking an Ocular Surface Squamous Neoplasia in a Young Adult Filipino: A Case Report and Literature Review

**DOI:** 10.1155/2024/3113342

**Published:** 2024-07-11

**Authors:** Lorenz Jacob Mangahas, Rowena Wea Reyes, Richmond Siazon

**Affiliations:** ^1^ Department of Ophthalmology Ilocos Training and Regional Medical Center, San Fernando, La Union 2500, Philippines; ^2^ Department of Laboratories Ilocos Training and Regional Medical Center, San Fernando, La Union 2500, Philippines

**Keywords:** basal cell carcinoma, conjunctival tumors, immunohistochemistry, ocular surface squamous neoplasia

## Abstract

**Objective:** To describe the morphological and histopathological features of primary conjunctival basal cell carcinoma (BCC) in a young adult Filipino.

**Introduction:** Malignant conjunctival tumors arise from different cells, the most common of which are squamous cell carcinomas (SCCs), (including ocular surface squamous neoplasia [OSSN]), melanomas, and lymphomas. Primary conjunctival BCC is rare and can mimic the clinical features of OSSN. Only seven reported cases were published. Most cases are in the 6th–8th decades of life, and we report the first case in a young adult male.

**Case Summary:** A 37/M, HIV-seronegative, presenting with a 3-year history of enlarging fleshy, pedunculated mass on the right eye measuring 8.5 mm × 8.0 mm at the superonasal limbus encroaching on the cornea, with prominent feeder vessels. Whitish-to-grayish plaques were observed on the surface of the lesions. Wide excision of the mass using the no-touch technique was performed under local anesthesia. Four cycles of mitomycin C (0.02%) were administered as chemoadjuvant therapy. Histopathology showed basaloid cells with peripheral palisading, most consistent with BCC. Immunohistochemistry was positive for Bcl-2 and CD10 markers and negative for epithelial membrane antigen (EMA) and carcinoembryonic antigen (CEA), confirming conjunctival BCC. Eight weeks postoperatively, fibrovascular tissue proliferation was noted at the excision site. Anterior segment-optical coherence tomography (AS-OCT) revealed a thickened hyperreflective band that was continuous with the epithelium, indicating possible recurrence. Resection with rush frozen section revealed fibrotic tissue that was negative for tumor cells. The bare sclera was covered with conjunctival autograft. There was no recurrence of the lesion after 16 months of follow-up.

**Conclusion:** Primary BCC of the conjunctiva is rarely encountered, especially in young individuals, mimicking squamous neoplasia both in morphology and histopathology. Therefore, this should be considered in the differential diagnosis of OSSN. Immunostaining is crucial in differentiating between the two conditions and confirming the diagnosis. In most cases, wide surgical excision is sufficient. In addition, adjuvant therapies may be beneficial in preventing tumor recurrence.

## 1. Introduction

Basal cell carcinoma (BCC) is the most common eyelid neoplasm, accounting for over 90% of all cases, and primarily affects cutaneous tissues. Mucous membranes, such as the conjunctiva, are uncommon predilection sites.

The most common malignant conjunctival tumors are squamous cell carcinoma (SCC), melanomas, and lymphomas [1, 2]. Primary conjunctival BCC is rarely observed and can mimic the clinical features of ocular surface squamous neoplasia (OSSN). According to some researchers, they may originate from metaplasia of pluripotent basal germ cells or, in some cases, pre-existing squamous papillomas or intraepithelial epithelioma [3–5]. Conjunctival BCC can also occur secondarily from eyelid or caruncular extension [6].

In the literature, there have been seven reported cases of primary BCC of the conjunctiva [4–10]. We describe the first case of a young adult male of Malay ethnicity with a purely conjunctival mass mimicking OSSN.

## 2. Case Presentation

A 37-year-old Filipino male presented with a progressively enlarging fleshy fibrovascular mass in his right eye, causing blurring of vision, foreign body sensation, redness, and occasional tearing. No eye pain was observed. He worked as a tricycle driver for more than a decade, with prolonged sun exposure and no history of eye trauma or surgery.

**Figure 1:**
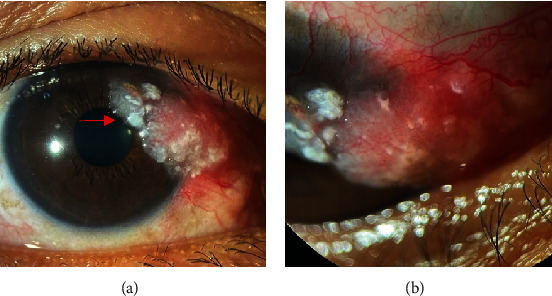
(a, b) An 8.5 × 8.0 mm elevated pedunculated, fibrovascular, fleshy mass at the superonasal limbus encroaching the cornea.

On examination, an 8.5 mm × 8.0 mm elevated, pedunculated, fibrovascular, fleshy mass at the superonasal limbus encroaching on the cornea of the right eye was observed (Figure 1). The surface of the lesion was irregular, with adherent whitish-to-grayish plaques typically seen in OSSN (Figure 1, arrow). Several feeder vessels surrounding the lesion can also be observed (Figure 1(b)). No other lesions were observed in the ocular adnexa. Dilated funduscopy was normal. No palpable cervical lymph nodes were observed. OSSN was the primary consideration owing to its clinical features and the young age of the patient. He is HIV-seronegative. A wide excision of the mass using a no-touch technique was performed under local anesthesia, and the specimen was sent for histopathological examination. The conjunctival bare sclera was treated with on-lay mitomycin 0.04% for 2 min, followed by copious irrigation with normal saline solution. The bare sclera was covered by advancement of the conjunctiva and secured in place with interrupted nylon 10-0 sutures. The patient underwent postoperative chemoadjuvant therapy with mitomycin C 0.02% QID, (1 week on, 1 week off), for four cycles. During the week off, the patient used sodium hyaluronate eye drops (QID). The patient was monitored at 2-week intervals.

**Figure 2:**
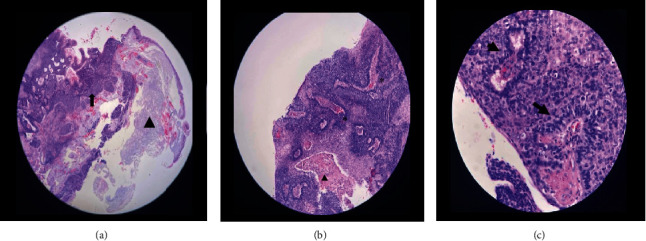
Microscopic section stained with H&E showing sheets of irregular papillae lined by basaloid cells arranged in peripheral palisading pattern. (a) Scanner view shows thickening of the epithelium with dysplastic cells occupying the full thickness of the conjunctival epithelium with nests of tumor cells extending to the underlying stroma (arrow) and an underlying solar elastosis (arrowhead). (b) LPO view showing lesions exhibiting papillary configuration in peripheral palisading pattern with intralesional fibrovascular core (∗) and are as of necrosis (arrowhead). (c) HPO view showing lesions exhibiting papillary configuration in peripheral palisading pattern with intralesional fibrovascular core (arrow).

**Figure 3:**
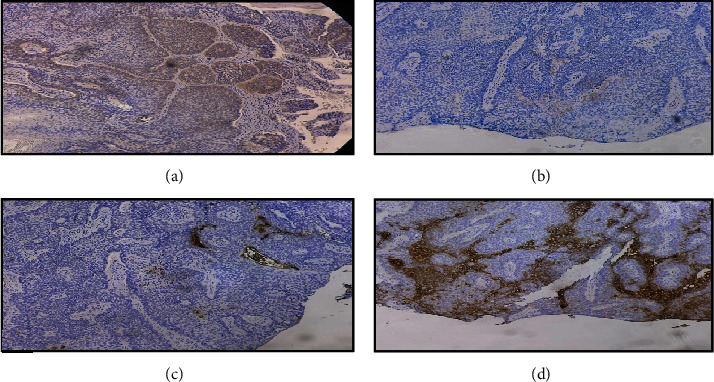
HPO view of (a) BCL2, (b) CD10, (c) EMA, and (d) CEA immunohistochemical stains. Positivity for BCL2 and CD10 stains was confirmed by the brown color of the neoplastic cells. EMA and CEA show negative expression on neoplastic cells.

**Figure 4:**
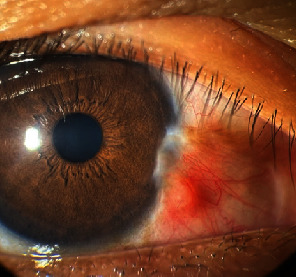
The patient at 8 weeks post excision of the conjunctival mass showing regrowth of the fibrovascular mass.

**Figure 5:**
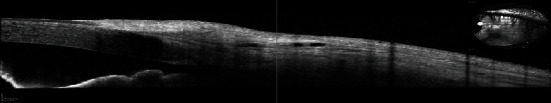
Anterior segment OCT of the recurrence of the conjunctival mass showing a hyperreflective band continuous with the conjunctival epithelium.

**Figure 6:**
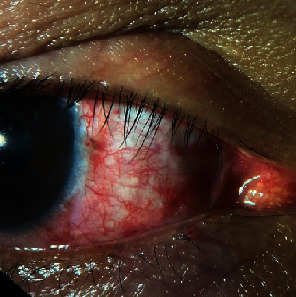
No recurrence noted at 6 months postoperatively.

The histopathologic report revealed soft gray tissue measuring 6 mm × 5 mm × 3 mm. Microscopically, basaloid cells with peripheral palisading were observed, consistent with BCC (Figures 2(a), 2(b), and 2(c)). The tumor cells were present at the basal margins. Immunohistochemistry was requested for confirmatory testing, which revealed positive Bcl-2 and CD10 markers (Figures 3(a) and 3(b)) and negative epithelial membrane antigen (EMA) and carcinoembryonic antigen (CEA) markers (Figures 3(c) and 3(d)). Fibrovascular tissue proliferation near the nasal limbus was noted at 8 weeks postoperatively, indicating possible recurrence (Figure 4). Anterior segment-optical coherence tomography (AS-OCT) was performed to assess the depth of the lesion. A hyperreflective band of the conjunctival mass that was continuous with the epithelium was noted (Figure 5). Surgical resection with rush frozen section revealed fibrotic tissue proliferation and the absence of tumor cells. The bare sclera was covered with conjunctival autograft. The patient was prescribed a topical antibiotic and steroid combination at home. No recurrence was noted 16 months postoperatively (Figure 6).

## 3. Discussion

BCC predominantly occurs on sun-exposed hair-bearing skin of the face, such as the eyelids and caruncle. The conjunctiva is an infrequent site for BCC. Only seven primary conjunctival BCCs have been published online. Of these, only two were confirmed positive by immunohistochemistry (Table 1).

Conjunctival BCCs are indolent with slow progression and tend to occur in men aged > 60 years. However, in our case, the patient was a relatively young adult male of Malay descent in his 30s.

The etiology and development of conjunctival BCC are not fully understood. It has been suggested that conjunctival BCCs originate from the metaplasia of pluripotent basal epithelial germ cells due to UV damage, resulting in adnexal type or epidermal-type epithelium that eventually advances to primary conjunctival BCC [3]. This finding may explain the tendency of this tumor to occur along the nasal and temporal limbal areas, where nests of corneal epithelial stem cells are most exposed to UV rays. All documented cases were found in the nasal and temporal limbus (Table 1). In our case, a noteworthy finding was the presence of solar elastosis alongside the tumor cells in the sections of the lesion (Figure 2(a)). Although still relatively young, our patient had significant sun exposure, which put him at high risk. Another study postulated that a dermoid choristoma in the conjunctiva, which was absent in our case, may lead to BCC [8]. Some authors also proposed that pre-existing squamous papillomas give rise to conjunctival BCC owing to the papillary appearance in some cases [3]. Furthermore, some reported cases are secondary to local seeding or extension from a previously excised adjacent BCC, which was not the case for our patient, who had no cutaneous or ocular adnexal lesions.

Interestingly, the morphologic features of documented cases vary from a fleshy, pedunculated lesion such as OSSN or a limbal nodule to a highly pigmented lesion mimicking a melanoma [4–10]. Hence, diagnosing conjunctival BCC can be challenging due to its morphologic variability and rarity. In our case, the fleshy irregular mass with white plaques on the lesion's surface is distinctive and has been previously described in one documented case [10].

It was further asserted that conjunctival BCC may arise from pre-existing leukoplakia [3].

Distinguishing BCC from other tumors such as SCC is crucial for proper diagnosis and management. The different immunostaining profiles of BCC and SCC can aid in distinguishing between them histologically. One recent case revealed positivity for p53, p63, CD10, and BCL2, favoring conjunctival BCC, while BerEP4 and EMA expression were negative [9]. However, another case exhibited positivity for BerEP4, which is typically seen in all cases of cutaneous BCC, but its presence in conjunctival BCC has yet to be established [10]. In our case, immunohistochemical staining was performed, showing positive expression of C10 and Bcl-2 in tumor cells and negative for CEA and EMA, confirming the diagnosis of conjunctival BCC. It was determined that positivity for Bcl-2 and CD10 markers had 88% accuracy and 100% specificity in detecting BCC, while CEA and EMA positivity detected SCC with 67% accuracy and 100% specificity [11].

Although treatment has not been firmly established, all reported cases were successfully treated with wide excision, except for one eye that was enucleated due to intraocular invasion [7]. A retrospective study by Gasiorowski et al. on the risk factors for orbital invasion of periocular tumors revealed that multifocal, large (21–30 mm), elevated lesions, and advanced age were associated with the likelihood of orbital invasion, hence requiring exenteration.

Globe-sparing surgeries for specific cases of single precious eye or anterior orbital involvement have been reported to be a good option, but annual MRI examinations for at least 5 years postsurgery are needed [12]. In this review of conjunctival BCC, the absence of recurrence after complete excision may suggest adequate management. In our case, no recurrence was observed 16 months postoperatively. Furthermore, adjuvant chemotherapy may be beneficial in preventing tumor recurrence; however, this requires further investigation.

## 4. Conclusion

Primary BCC of the conjunctiva is rare. However, it should be considered as a differential diagnosis of OSSN, especially in young individuals with a history of significant UV-sun exposure. Clinical, morphological, and histopathologic features may resemble typical OSSN; as such, the use of immunohistochemistry may be necessary for definitive diagnosis. Complete surgical excision may be sufficient based on documented cases; however, the use of adjunctive therapies could help prevent tumor recurrence.

## Figures and Tables

**Table 1:** Summary table of published case reports on primary conjunctival basal cell carcinoma.

**Author (year)**	**Age/sex**	**Location**	**IHC ** ^a^	**Treatment**	**Follow-up data**
Aftab and Percival (1973)	82 M	Nasal	No	Excision	No recurrence after 2 months
Apte (1975)	69 F	Nasal	No	Excision	No recurrence
Husain (1993)	66 M	Nasal	No	Excision	No recurrence after 12 months
Cable (2000)	69 M	Temporal	No	Enucleation	No systemic metastasis
Mudhar (2020)	60 M	Temporal	No	Wide excision with free margins, double-freeze cryotherapy with MMC	No recurrence after 2 months
Low (2022)	67 M	Temporal	Yes^b^	Wide excision with free margins, double-freeze cryotherapy with MMC	No recurrence after 3 months
Lin (2023)	73 M	Temporal	Yes^c^	Excision	No recurrence after 6 months
Present case (2023)	37 M	Nasal	Yes^d^	Wide excision; repeat excision of the mass regrowth with RFS	No recurrence after 16 months

*Note:* Adapted from a table by Lin, Hong, and Harocopos [10].

^a^Immunohistochemistry.

^b^IHC exhibited +BerEP4, +BCL2, -CK20, -EMA, and -TTF1.

^c^IHC exhibited -BerEP4, -CK7, -EMA, -S100, and +p63.

^d^IHC exhibited +Bcl-2, +CD10, -EMA, and -CEA.

## Data Availability

The data used to support the findings of this study are included within the article.
